# Growth and Characterization of n-Type Hexagonal Ta_2_O_5_:W Films on Sapphire Substrates by MOCVD

**DOI:** 10.3390/ma18133073

**Published:** 2025-06-28

**Authors:** Xiaochen Ma, Yuanheng Li, Xuan Liu, Deqiang Chen, Yong Le, Biao Zhang

**Affiliations:** 1 Key Laboratory of Optoelectronics Technology, School of Information Science and Technology, Beijing University of Technology, Beijing 100124, China; liyuanheng@emails.bjut.edu.cn (Y.L.); realiuxuan@emails.bjut.edu.cn (X.L.); chendeqiang@emails.bjut.edu.cn (D.C.); 2 School of Information and Electronic Engineering, Shandong Technology and Business University, Yantai 265600, China; 3 School of Integrated Circuits, Shandong University, Jinan 250101, China; 202032491@mail.sdu.edu.cn

**Keywords:** *δ*-Ta_2_O_5_, W doping, structural characterization, electrical properties, X-ray diffraction, TEM

## Abstract

Tantalum oxide is a wide bandgap material commonly used as an insulating dielectric layer for devices. In this work, hexagonal Ta_2_O_5_ (*δ*-Ta_2_O_5_) films doped with tungsten (W) were deposited on *α*-Al_2_O_3_ (0001) by metal–organic chemical vapor deposition (MOCVD). The effects of W doping on the structural, morphology, and photoelectrical properties of the obtained films were studied. The results showed that all W-doped films were n-type semiconductors. The XRD measurement result exhibited that the increase in the W doping concentration leads to the changes in the preferred growth crystal plane of the films from *δ*-Ta_2_O_5_ ($10\bar{1}1$) to (0001). The 1.5% W-doped film possessed the best crystal quality and conductivity. The Hall measurement showed that the minimum resistivity of the film was 2.68 × 10^4^ Ω∙cm, and the maximum carrier concentration was 7.39 × 10^14^ cm^3^. With the increase in the W concentration, the surface roughness of the film increases, while the optical bandgap decreases. The optical band gap of the 1.5% W-doped film was 3.92 eV. The W doping mechanisms were discussed.

## 1. Introduction

Tantalum pentoxide (Ta_2_O_5_) is a wide bandgap, high resistance, and high-k material with a dielectric constant of approximately ~22. Its bandgap of about 4.0 eV is much wider than that of gallium nitride (3.4 eV) and silicon carbide (3.3 eV), resulting in a significant high breakdown electric field of 7 MV·cm^−1^, which is more than twice that of gallium nitride and silicon carbide [1,2]. Attributed to its high breakdown electric field and low leakage current, Ta_2_O_5_ films are commonly used as the gate dielectric layer for devices such as thin film transistors [3,4,5,6], high electron mobility transistors [7,8,9,10,11], and MOSFET [12] to reduce the threshold voltage and power consumption [13].

Ta_2_O_5_ also has been widely studied and applied in many fields, including photocatalysis [14,15,16], super capacitors [17,18], gas sensing [19,20,21], and memory devices [22,23,24]. In addition, the research on Ta_2_O_5_ is constantly expanding in fields such as optics [25], biomedical detectors [26], medical treatments [27], microbiology [28], and environmental monitors [29]. Some research has been conducted on tantalum oxides doped with different dopants, such as S, N, Al, and Eu, to improve their photocatalytic efficiency, dielectric properties, and photoresponsivity. Ismail synthesized mesoporous sulfur-doped Ta_2_O_5_ nanocomposites and studied their photocatalytic activities [30]. Shi investigated the adsorption and visible-light catalytic activity of N-doped Ta_2_O_5_ nanomaterials [31]. Spassov studied the Al-doped Ta_2_O_5_ prepared by sputtering and revealed that the dopant Al can increase the dielectric constant and suppress the deep oxygen vacancy centers in tantalum oxide [32]. Miura observed the blue emission band of Eu-doped tantalum oxide thin films prepared by the co-sputtering method [33].

The excellent properties of the wide bandgap and high dielectric constant indicate that tantalum oxide has the potential to become a new type of high-performance wide bandgap oxide semiconductor material. However, only a few reports focused on improving the conductivity of tantalum oxide through doping [34]. Due to the lack of research on the electrical properties of tantalum oxide currently, it is mostly not used as a semiconductor active layer material.

The growth and effective doping of tantalum oxide thin films on commonly used substrates are important foundations for the application of tantalum oxide as a wide bandgap semiconductor active layer material. The *α*-Al_2_O_3_ wafer is an ideal substrate since it is one of the most commonly used substrates in many fields due to its high stability, low prince, and its hexagonal lattice structure, and it is highly compatible with GaN. A proper dopant is indispensable for effective doping. In order to realize the donor energy level by the substitutional doping of tantalum ions (Ta^5+^), the valence state of the dopant ion should be greater than +5. To maintain a high crystalline quality, the radius of the dopant should be close to that of Ta^5+^ (0.064 nm). In this case, the tungsten ion (W^6+^) with a valence state of +6 and a radius of 0.06 nm is chosen as the dopant. In addition, when tungsten exists in lattice gaps, donor energy levels should also be formed. Our previous research has demonstrated the feasibility of achieving n-type Ta_2_O_5_ films through W doping [35].

In this paper, W-doped Ta_2_O_5_ (Ta_2_O_5_:W) films are deposited on *α*-Al_2_O_3_ (0001) substrates by MOCVD. The Ta_2_O_5_:W thin films with the W/Ta atomic ratios of 0%, 0.5%, 1.5%, 2.5%, and 3.5% are deposited. We systematically explored the effects of the doping concentration on the lattice, electrical, and optical characteristics of thin films.

## 2. Materials and Methods

### 2.1. Equipment and Materials

The W-doped Ta_2_O_5_ films were deposited using stainless steel, cold-wall, dual-flow-structured, and high-vacuum MOCVD equipment [36]. The double-side polished *α*-Al_2_O_3_ (0001) single crystal wafers with a thickness of 0.5 mm were used as substrates. Tantalum ethoxide (TaC_10_H_25_O_5_) and hexacarbonyl tungsten [W(CO)_6_] were used as MO sources for tantalum and tungsten, respectively, and placed in stainless steel bottles. TaC_10_H_25_O_5_ is a volatile liquid at room temperature (vapor pressure at 25 °C: 82.8 mm Hg), while W(CO)_6_ is a volatile solid (vapor pressure at 67 °C: 1.2 mm Hg). By tuning the temperature, pressure, and carrier gas flow rate of the MO source steel bubbler, the molar flow rate of the MO source was controlled. High-purity oxygen O_2_ (5N) was used as an oxidant. The precursor and oxidant are transported to the reaction chamber through independent pipelines by ultra-high-purity N_2_ (9N).

### 2.2. Process Parameters

The setting of the film preparation conditions referred to our previous growth of the intrinsic *δ*-Ta_2_O_5_ films on *α*-Al_2_O_3_ (0001) substrate [37]. The reaction chamber pressure was fixed at 20 Torr. The samples were placed on the sample plate in the chamber, and the plate was heated to a high temperature of 750 °C. O_2_ was delivered into the chamber at a flow rate of 50 sccm during the film preparation process. The temperature and pressure of the ethanol tantalum source bottle were 90 °C and 20 Pa, respectively, and tantalum ethoxide was sent to the chamber at a fixed molar flow rate of 3.96 × 10^−6^ mol·min^−1^. The temperature and pressure of the hexacarbonyl tungsten source bottle were 20 °C and 290 Pa, respectively. In order to obtain W doping concentrations of 0%, 0.5%, 1.5%, 2.5%, and 3.5%, by adjusting the gas flow rate into the steel bubbler from 0 to 21 sccm, the hexacarbonyl tungsten was sent to chamber at the different molar flow rates from 0 to 1.4 × 10^−7^ mol·min^−1^, respectively, and the corresponding samples were marked as S0, S1, S2, S3, and S4. The growth process of the film lasted for five hours. The detailed deposition parameters and result are shown in Table 1.

### 2.3. Characterization Methods

In order to determine the structure properties of the tantalum oxide films, X-ray diffraction (XRD) *θ*–2*θ* scan and *ω*-rocking curve were performed by an XRD diffractometer (Rigaku smartlab 3 kw (Rigaku, Akishima, Japan)). The surface topography and roughness of the films were obtained by atomic force microscopy (AFM, BioScope Resolve (Bruker, Billerica, MA, USA)). High-resolution transmission electron microscopy (HRTEM, FEI Talos F200X-G2 (Thermo Fisher Scientific, Waltham, MA, USA)) measurements were conducted to identify the orientation relationship and microstructure of the Ta_2_O_5_:W films. The optical transmission spectra of the samples were measure by a double beam UV–Vis–NIR spectrophotometer (TU-1901) (Pgeneral, Beijing, China), and the optical bandgaps of the films were determined. X-ray photoelectron spectroscopy (XPS, Thermo Scientific ESCALAB Xi+ (Thermo Fisher Scientific, Waltham, MA, USA)) was performed to analyze the chemical composition and bonding state of the samples. The Hall electrical properties of the films were measured using the Van der Pauw method (M91-HR, Lake Shore (Carson, CA, USA)) at room temperature with 0.5 Tesla and 10^−7^ Ampere.

## 3. Results and Discussion

The film thicknesses of the deposited samples, S0, S1, S2, S3, and S4, are about 208, 220, 260, 258, and 257 nm, respectively. As the W concentration increases, the thickness of the film first increases and reaches its maximum value at a 1.5% W concentration, then slightly decreases. The lattice structures, growth orientation, and crystal quality of the samples were characterized and analyzed by XRD.

**Figure 1:**
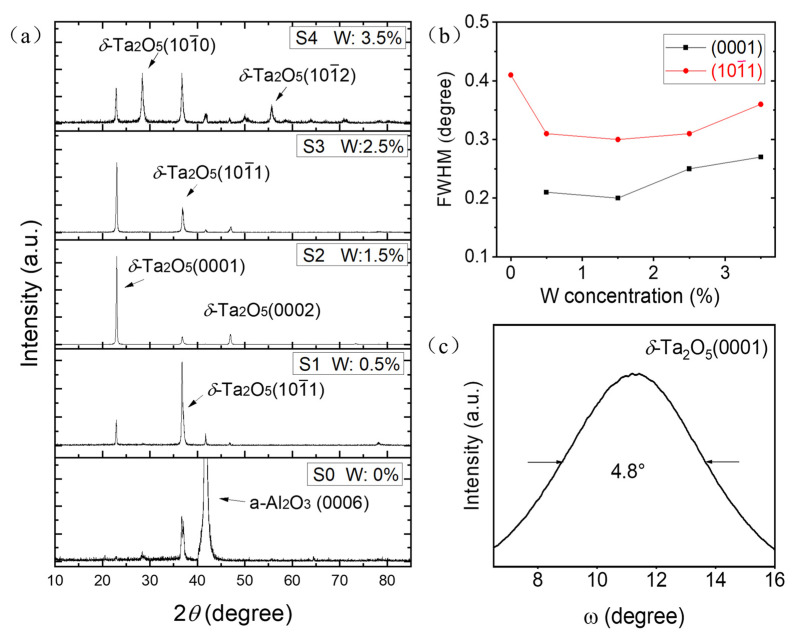
(**a**) The XRD *θ*–2*θ* scan spectra of the samples S0–S4, (**b**) the FWHM of (0001) and ($10\bar{1}1$) peaks as a function of the W doping concentration, and (**c**) the *ω*-rocking curve of the *δ*-Ta_2_O_5_ (0001) peak for sample S2 with a W doping concentration of 1.5%.

Figure 1a exhibits the XRD *θ*–2*θ* scan spectra of the samples S0–S4 with W doping concentrations of 0%, 0.5%, 1.5%, 2.5%, and 3.5%, respectively. In the spectra, the diffraction peak near 41.65° corresponds to the *α*-Al_2_O_3_ (0006) plane (JCPDS 10-0173). For the spectrum of S0, besides the signal of the substrate, only one peak near 36.7° appears, which corresponds to the hexagonal phase *δ*-Ta_2_O_5_ ($10\bar{1}1$) plane (JCPDS 19-1299). The result is consistent with our previous reports on intrinsic films [37]. For sample S1, besides the *δ*-Ta_2_O_5_ ($10\bar{1}1$) peaks, another two peaks located at 22.8° and 45.6° can be observed, corresponding to the *δ*-Ta_2_O_5_ (0001) and (0002) plane, respectively, implying that the film is polycrystalline *δ*-Ta_2_O_5_ with two different orientations. According to the Bragg formula and the diffraction peak angles of Ta_2_O_5_ (0001) and ($10\bar{1}1$), the lattice parameters are calculated as a = 0.3630 nm and c = 0.3897 nm, which are consistent with the results on the JCPDS card. For the spectrum of S2 (W:1.5%), the *δ*-Ta_2_O_5_ (0001) peak becomes very strong, while the ($10\bar{1}1$) peak becomes weak, indicating that the preferred orientation of the film has changed to *δ*-Ta_2_O_5_ [0001]. For the spectrum of S3, the results are similar to those of S2, except for a significant enhancement in the ($10\bar{1}1$) peak. For sample S4, *δ*-Ta_2_O_5_ (0001), ($10\bar{1}0$), ($10\bar{1}1$), and ($10\bar{1}2$) peaks can be observed, implying that the film is a single-phase polycrystalline *δ*-Ta_2_O_5_ without a preferred orientation. The full-width at half maximum (FWHM) of the (0001) and ($10\bar{1}1$) peaks as a function of the W doping concentration is shown in Figure 1b. The trends of the FWHM values of the two peaks are the same. They decrease firstly and then increase with the increase in the W doping concentration. The 1.5% W-doped film exhibits the lowest FWHM, with corresponding values of 0.2 and 0.3°, implying that the crystallization quality of the 1.5% W-doped film is the best. The lattice constant of the *δ*-Ta_2_O_5_ is a_t_ = 0.3624 nm, which is smaller than that of the *α*-Al_2_O_3_ (a_a_ = 0.4758). For the *δ*-Ta_2_O_5_ (0001) plane on sapphire in the c-plane, if four cycles of a_t_ are grown from three cycles of a_a_, the lattice mismatch rate is about 1.5%, and the tantalum oxide film is under compressive stress. The ionic radius of the W^6+^ is 0.060 nm, which is smaller than that of Ta^5+^ by 0.064 nm. Therefore, when replacing Ta^5+^ ions with W^6+^ ions, the interplanar spacing of *δ*-Ta_2_O_5_ will decrease, which reduces the mismatch and leads to the transition of the preferred growth crystal plane from *δ*-Ta_2_O_5_ ($10\bar{1}1$) to (0001). A relatively low FWHM value indicates that the obtained polycrystalline film has a good crystalline quality. Figure 1c exhibits the XRD *ω*-rocking curve of the *δ*-Ta_2_O_5_ (0001) peak for the S2 sample with W doping concentrations of 1.5%. The peak shape is symmetrical, with a FWHM value of 4.8°. Although the FWHM is relatively wide, it indicates that the crystalline quality is significantly better than that of ordinary polycrystals.

The average grain size can be determined by the Scherrer formula D = 0.89λ/βcos*θ*, where D is the grain size, *λ* is the wavelength of the X-ray, β is the FWHM of the diffractive peaks, and *θ* is the diffractive angle [38]. The grain size of the films for samples S0–S4 were calculated to be 20.2, 26.7, 41.4, 32.1, and 23.0 nm, respectively. It is closely related to the crystal quality, and the film doped with the 1.5% W has the largest grain size. The trend of the changes in the grain size is similar to that of the film thickness, indicating that the growth rate of the film is also related to the quality of the crystallization.

**Figure 2:**
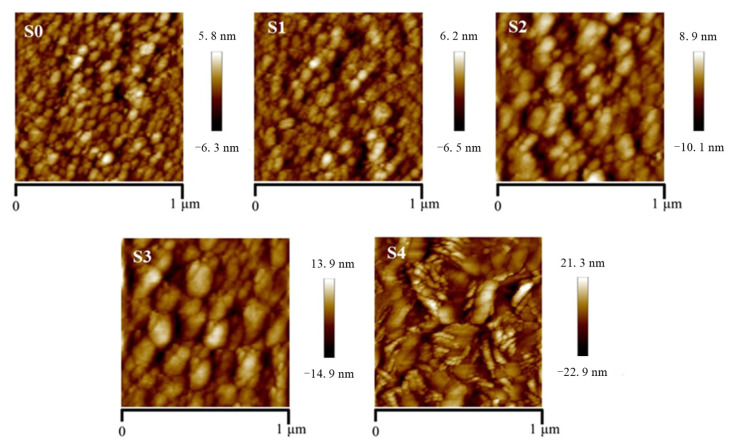
The AFM images of S0–S4 samples with a scanning area of 1 μm × 1 μm.

The AFM surface morphology images of the S0–S4 samples are shown in Figure 2, with a scanning area of 1 μm × 1 μm for each sample. The surface of samples S0 and S1 are relatively flat, and the surface grain size gradually increases with the increase in the W doping concentration. For samples S0 to S4, the measured root mean square (RMS) roughness is 1.66, 1.72, 2.77, 3.99, and 6.03 nm, respectively. The AFM results demonstrate that with the gradual increase in the W doping concentration, the surface roughness of the *δ*-Ta_2_O_5_:W film increases monotonically. Excessive doping concentrations can significantly affect the surface morphology and roughness of the film. According to the analysis results of the XRD, increasing the doping concentration simultaneously affects the grain size and lattice orientation, leading to an increase in the surface roughness of the film.

**Figure 3:**
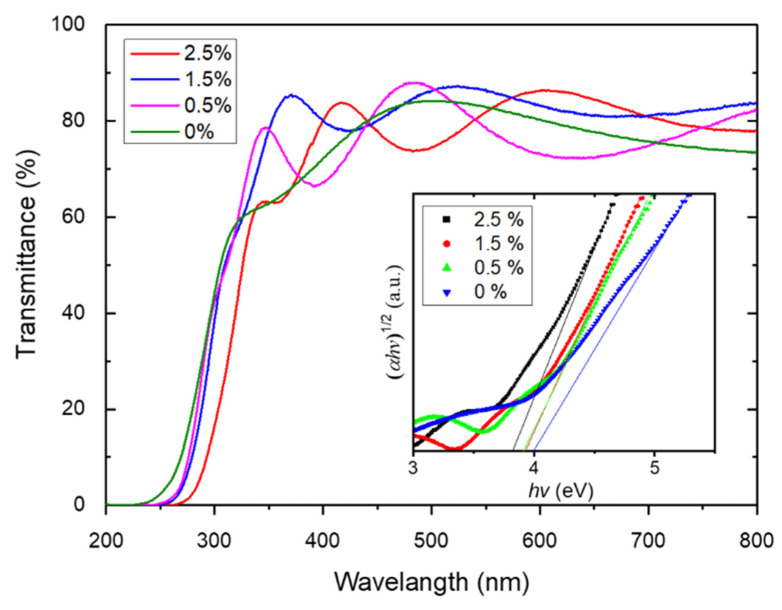
Optical transmission spectra of the S0–S3 samples with their (*αhν*)^1/2^-*hν* in the inset.

The optical transmission spectra of the samples are exhibited in Figure 3. The average transmittance of the S0–S3 samples in the visible light region is 78.8%, 77.8%, 82.7%, and 80.7%, respectively. The S4 sample appears slightly white, like frosted glass. A large surface roughness leads to an enhanced diffuse scattering of light and a reduced transmittance, which is consistent with the results of the AFM. The S2 sample shows the highest transmittance due to its high crystalline quality, large grain size, and regular lattice orientation. The Tauc formula A(*hν*−*E*_g_)^2^ = *αhν* is commonly used to estimate the optical band gap of a δ-Ta_2_O_5_ film due to its indirect bandgap [39], where A is a constant, *hν* is the energy of the photons, *E*_g_ is the optical band gap, and α is the absorption coefficient. The inset of Figure 3 shows the curves of the (*αhν*)^1/2^ against the *hν* derived from the optical transmission spectra. The optical band gaps of the films doped with W concentrations of 0%, 0.5%, 1.5%, and 2.5% are 4.01, 3.93, 3.92, and 3.83 eV, respectively. It can be seen that the optical band gap of the *δ*-Ta_2_O_5_:W films decreases with the W doping concentrations’ increase. The decrease in *E*_g_ can be ascribed to the narrower band gap of WO_3_ (~2.7 eV) [40] than that of Ta_2_O_5_, similarly to Sn-doped Ga_2_O_3_ films [41].

**Figure 4:**
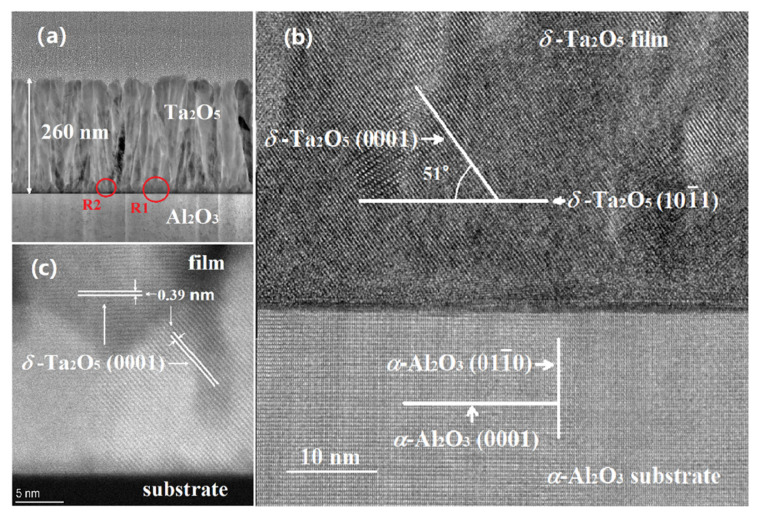
(**a**) The LRTEM image of the cross-section of sample S2. (**b**,**c**) The HRTEM photographs of labeled regions R1 and R2 at the *δ*-Ta_2_O_5_/*α*-Al_2_O_3_ interface.

The TEM image of the S2 sample (1.5% W doping) is exhibited in Figure 4. The low-resolution TEM (LRTEM) photograph shown in Figure 4a displayed a clear and smooth interface of the film–substrate. The thickness of the film is about 260 nm. The HRTEM micrograph of the R1 region is shown in Figure 4b. In the sapphire substrate area, the Al_2_O_3_ (0001) and ($01\bar{1}0$) crystal planes are marked. In the film region, the lattice arrangement of *δ*-Ta_2_O_5_ is regular and orderly, and the *δ*-Ta_2_O_5_ ($10\bar{1}1$) and (0001) planes as well as their intersection angles (51.1°) are marked, respectively. Among them, the *δ*-Ta_2_O_5_ ($10\bar{1}1$) plane is parallel to the substrate surface. This is consistent with the results of intrinsic films [37]. However, the micrograph of the film/substrate interface in the R2 area displays a different scene, as shown in Figure 4c. The *δ*-Ta_2_O_5_ (0001) planes along two different directions in different grains are marked in the figure, one of which is parallel to the substrate surface. From the TEM results, the 1.5% W-doped *δ*-Ta_2_O_5_ film is a polycrystalline with only two growth plans of ($10\bar{1}1$) and (0001), which is consistent with the XRD results.

**Figure 5:**
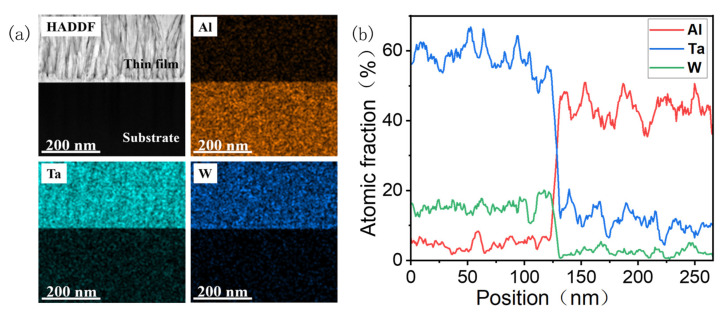
(**a**) EDS images of the *δ*-Ta_2_O_5_:W/Al_2_O_3_ interface, and (**b**) the EDS line scanning curve from the Ta_2_O_5_ film to the substrate.

Figure 5a shows the EDS mapping image of the film/substrate interface of the S2 samples. Among them, Ta, W, and Al elements are evenly distributed, and the interface surface is clearly visible. The EDS line scanning curves of each element content perpendicular to the *δ*-Ta_2_O_5_/*α*-Al_2_O_3_ interface are shown in Figure 5b. For all the detected elements, their content changes sharply at the interface, although the content of W is relatively low. These results demonstrate that the element diffusion at the interface is not severe.

**Figure 6:**
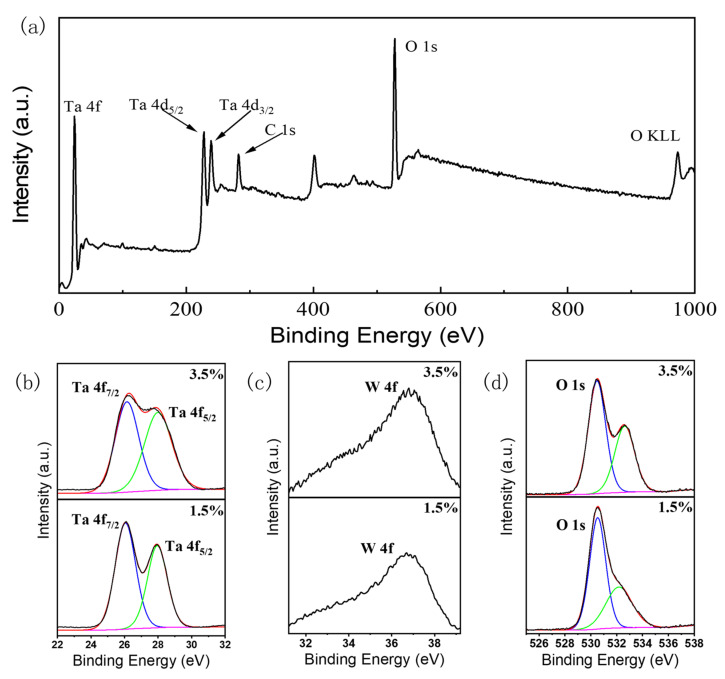
XPS spectra of the W-doped films: (**a**) a survey of sample S2 and fine spectra of (**b**) Ta 4f, (**c**) W 4f, and (**d**) O 1s for S2 and S4.

The XPS measurements were conducted to study the valence states of elements and W doping levels. In order to reduce the influence of the interface adsorption on the test results, the sample was etched about 10 nm by argon plasma before testing. The XPS survey spectrum of sample S2 is shown in Figure 6a. The C 1s, O 1s, Ta 4f, Ta 4d, and O KLL signal peaks are observed in the survey spectrum. To compare the samples with the highest crystal quality and the highest doping concentration, the Ta 4f, W 4f, and O 1s fine spectra of S2 and S4 are displayed in Figure 6b–d, respectively. In Figure 6b, two Gaussian fitting peaks located at 26.10 and 27.95 eV correspond to Ta 4f_7/2_ and Ta 4f_5/2_ core levels, respectively [42,43]. The W 4f signal peaks of S2 and S4 around 37.0 eV are observed in Figure 6c [44,45]. The XPS results confirm that the valence states of W and Ta ions in the film are mainly +6 and 5+, respectively. Additional electrons can be released to the conduction band by replacing Ta^5+^ with W^6+^. Figure 6d displays the two Gaussian fitting O 1s peaks located at 530.55 eV and 532.25 eV, which can be attributed to the lattice oxygen and surface-adsorbed oxygen of the *δ*-Ta_2_O_5_ film [46,47]. The higher adsorption oxygen peak in the S4 spectrum may be due to the oxygen adsorption at the grain boundaries and rough surfaces of the polycrystalline film. According to the atomic sensitivity factor method [48], for sample S2, the percentages of O, Ta, and W in the total number of atoms are 70.84%, 28.69%, and 0.46%, respectively. For sample S4, the percentages of O, Ta, and W in the total number of atoms are 70.56%, 28.26%, and 1.08%, respectively. The atomic ratios of Ta/O of the S2 and S4 sample were confirmed to be approximately 40.5% and 40.2%, respectively, which are lower than 42.0% of intrinsic films [37], as expected. The W doping concentrations of S2 and S4 are about 1.6% and 3.8%, respectively. The defect equation of the W^6+^ doping can be considered as $2WO_{3}\overset{Ta_{2}O_{5}}{\to }\;2W_{Ta}^{.}+2e^{\prime }+5O_{O}^{X}+O_{2}\left(g\right)/2$, where $W_{Ta}^{.}$ is the W ion occupying the site of the Ta ion with one positive charge. $O_{O}^{X}$ is the oxygen element in its position without any net charge. The defects formed by the replacement of the Ta^5+^ lattice site with the W^6+^ ion offers free electrons to realize the electronic performance.

**Figure 7:**
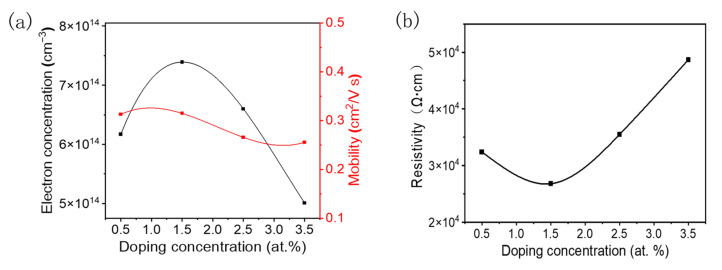
The variation in electrical properties of the films with W doping concentration: (**a**) Hall mobility and carrier concentration and (**b**) resistivity.

Due to the high resistance of S0, its Hall measurement results were not obtained. Figure 7 shows the variation in the electron Hall mobility, carrier concentration, and resistivity of *δ*-Ta_2_O_5_:W films with the W doping concentration. The results showed that as the W concentration increased from 0.5% to 3.5%, the Hall mobility slightly increased from 0.31 to 0.32 cm^2^∙V^−1^∙s^−1^ and then decreased to 0.26 cm^2^∙V^−1^∙s^−1^. Similarly, the carrier concentration initially increases from 6.17 × 10^14^ to 7.4 × 10^14^ cm^−3^ and then decreases to 5.0 × 10^14^ cm^−3^, with the maximum value occurring in the 1.5% W-doped film. The trend of the resistivity change is exactly the opposite. When the W doping level increases from 0.5% to 1.5%, the resistivity decreases from 3.24 × 10^4^ to a minimum value of 2.68 × 10^4^ Ω∙cm. As the doping concentration further increases to 3.5%, the resistivity increases to 4.87 × 10^4^ Ω∙cm.

Due to its high valence state, the W element doped into the Ta_2_O_5_ thin film acts as a donor, releasing additional valence electrons either as a substitute ion or interstitial atom, leading to the increased carrier concentration. The initial increase in the carrier mobility with the W content is attribute to the improvement of the crystalline quality of the film, and the subsequent decline is due to the decrease in crystal quality and the enhancement of the defect scattering. Attributed to the high crystal quality, large grain size, consistent lattice orientation, and appropriate doping concentration, the sample with the 1.5% W doping concentration displayed the highest mobility and carrier concentration, as well as the lowest resistivity. For W doping above 1.5%, the decrease in the carrier concentration is mainly caused by the reduction in the crystal quality and doping efficiency. As the W concentration further increases, a large number of defects, including ionized impurities and interstitial atoms, appear. In this case, when the W doping exceeds 1.5%, the degradation of the crystal quality and the enhancement of the carrier scattering lead to a decrease in carrier mobility and an increase in resistivity. The measurement results indicate that W can serve as a donor dopant for tantalum oxide films. Compared with a single-crystal thin film grown epitaxially on YSZ, the polycrystalline thin film on sapphire exhibits a relatively low mobility and rough surface [35]. Therefore, further research will focus on improving crystal quality and unifying the crystal direction to improve the performance of the material.

By introducing the W element as the dopant, n-type tantalum oxide films were successfully prepared on sapphire substrates. Compared with GaN and SiC, Ta_2_O_5_ exhibits a wide bandgap, high dielectric constant, high breakdown voltage, high chemical inertness, and high thermal stability, which are desired for applications in high-pressure, high-temperature, and highly chemically corrosive environments. The successful deposition of Ta_2_O_5_ on sapphire substrates also demonstrates its potential compatibility with GaN devices.

## 4. Conclusions

The *δ*-Ta_2_O_5_:W films with W concentrations varying from 0 to 3.5 at.% were deposited on *α*-Al_2_O_3_ (0001) substrates by MOCVD. The growth orientation of hexagonal films changes with the W doping concentration. The crystal quality of the 1.5% W-doped film is the best with the preferred growth crystal plane of *δ*-Ta_2_O_5_ (0001), while the 3.5% W-doped film is polycrystalline without a preferred orientation. The RMS roughness of the obtained films increases from 1.66 to 6.03 nm when the W concentrations increase from 0 to 3.5 at.%. Room-temperature Hall measurements indicate that all the W-doped films are n-type semiconductors. The variation ranges of the carrier concentration and resistivity are 5.0 × 10^14^–7.4 × 10^14^ cm^−3^ and 2.7 × 10^4^–4.9 × 10^4^ Ω∙cm, respectively. The optical band gap of the *δ*-Ta_2_O_5_ films decreases from 4.01 to 3.83 eV with the increase in the W concentration from 0 to 3.5 at.%. The n-type *δ*-Ta_2_O_5_ films doped with tungsten can be used as a semiconductor active layer, which not only expands the application field of tantalum oxide in semiconductor devices but also has a wide range of application prospects.

As a newly researched wide bandgap material, further research will be conducted on its performance improvement (mobility and carrier concentration), doping activation, and metal semiconductor contact. After improving its performance, tantalum oxide is expected to be used for ultraviolet detectors and high-voltage power devices in extreme environments.

## Figures and Tables

**Table 1:** Deposition parameters for samples with different doping concentrations.

Sample No.	Mo Source	Bubbler Temp. (°C)	Bubbler Pressure (Torr)	Carrier Gas Flow Rate (sccm)	Molar Flow Rate (mol/min)	Film Thickness (nm)	Deposition Rate (nm/min)
S0–S4	Ta	90 °C	20	45	3.96 × 10^−6^	208	0.693
S1	W	20 °C	290	3	2.00 × 10^−8^	220	0.733
S2	W	20 °C	290	9	6.00 × 10^−8^	260	0.867
S3	W	20 °C	290	15	1.00 × 10^−7^	258	0.86
S4	W	20 °C	290	21	1.40 × 10^−7^	257	0.857

## Data Availability

The original contributions presented in this study are included in the article. Further inquiries can be directed to the corresponding authors.
